# *Clostridium* epsilon toxin is excessive in multiple sclerosis and provokes multifocal lesions in mouse models

**DOI:** 10.1172/JCI169643

**Published:** 2023-05-01

**Authors:** Anthony T. Reder

**Affiliations:** Department of Neurology, University of Chicago Medicine, Chicago, Illinois, USA.

## Abstract

Multiple sclerosis (MS) is an inflammatory disease of the CNS. In this issue of the *JCI*, Ma and Sannino et al. show that two strains of intestinal *Clostridium perfringens*, known to produce epsilon toxin (ETX), were frequently found in patients with MS. Tiny amounts of this toxin added to immunization with myelin antigens provoked MS-like brain lesions in mice. The distribution of these lesions was diffuse, as in MS, in contrast to the spinal cord–restricted lesions of most animal models. ETX bound to endothelial cells of the CNS to enhance immune cell trafficking through the blood-brain barrier into inflammatory brain lessons. ETX also binds to human, but not murine, white blood cells, perhaps altering immune responses. Barrier disruption and changes in immunity due to the toxin could alter the benefits of immune-modulatory MS therapies and are likely to interact with the complex genetics and environmental influences seen in MS.

## Brain inflammation without a cause describes multiple sclerosis

Multiple sclerosis (MS) is an inflammatory disease of the brain and spinal cord. The genesis of MS is complex, with multiple virus and CNS abnormalities, but without a definitive origin. Genetic underpinnings are exemplified by 200 MS-linked SNPS and nearly 9,000 dysregulated genes that are expressed in blood mononuclear cells in MS compared with healthy controls (1, 2). Immune contributions are reflected in fluctuating peripheral Th1 cell and B cell immune activation coincides with reduced CD8^^+^^ and CD4^^+^^ Treg function and subnormal type I interferon levels, leading to macrophage- and cytolytic T cell–mediated CNS demyelination (3, 4, 5). Clinical features include frequent, seemingly spontaneous lesions that appear in different areas of brain and spinal cord causing a myriad of symptoms. Disease complexity is also revealed in the number of therapies; 22 immune-modifying therapies reduce MS attacks by employing nine very different mechanisms of action. Environmental influences are reflected in the onset and exacerbations, which are both amplified by smoking, low vitamin D levels, Western/industrialized diet, and obesity; females also have a greater risk of MS. Although patients with MS have 50% fewer virus infections than people in the healthy control group, bacterial infections and upper respiratory virus infections can trigger attacks, and Epstein-Barr virus seems to provoke onset of MS in susceptible individuals (6, 7). Notably, rodent models do not recapitulate MS. Experimental autoimmune encephalomyelitis (EAE) is typically antigen-induced, often monophasic, and is restricted to the spinal cord. However, MS has no known antigen, and recurrent CNS damage affects the spinal cord and brain. Regardless, both diseases are governed by genetics, aging, immune variation, CNS repair capacity, and environment.

The gut microbiome appears to affect MS. In the context of MS, proinflammatory, Th1-inducing bacterial taxa, such as *Acinetobacter*, which are usually rare, are overrepresented, and antiinflammatory Th2-inducing taxa are underrepresented. *Acinetobacter* induces human IFN-γ–secreting T cells and reduces CD4^^+^^ Tregs in culture. Supporting a proinflammatory effect, fecal transplants from patients with MS worsen EAE (8). However, the gut bacteria, viruses, or toxins associated with MS, and the mechanisms that lead to inflammation or disruption of the blood-brain barrier (BBB), remain unknown.

## *Clostridium perfringens* epsilon toxin in MS and EAE

Ma et al. (9) report that 74% of patients with relapsing remitting MS (RRMS) harbored *Clostridium perfringens* within the small intestine microbiome, compared with 45% of matched people in the healthy control group. The infection was seemingly benign and without enteritis. Demonstrating an important consequence of the altered MS gut microbiome, the authors showed that 40% of MS stool samples, compared with only 0.001% of samples from people in the healthy control group, contained *C*. *perfringens* type B and D strains that produce epsilon toxin (ETX). This finding arose from the use of an improved isolation technique that captured bacterial signatures from the small intestine and not just the colon; the authors also employed sensitive PCR to accurately quantitate DNA reflective of bacterial abundance.

The ETX monomer crosses the small intestine without causing overt injury or enteritis, but it is associated with slowed GI motility and more mucosal tight junction permeability. The ETX receptor, myelin and lymphocyte protein (MAL), is restricted to endothelial cells in the CNS. MAL also appears on mature oligodendroglia and peripheral nerve Schwann cells, but not on oligodendrocyte precursors, other glia, or neurons. MAL is present on mature human T cells, but not on murine T cells, creating a human-specific effect on immunity.

Participants in Ma et al.’s study had not been exposed to antibiotics for more than six months. A possible cofounder in 22 of 31 patients was use of immunomodulating therapies (9). This detail is relevant because interferon-β therapy normalizes the disrupted microbiome in MS (10), and other MS therapies inhibit secretion of ETX in vitro (11).

## “Good fences make good neighbors,” Robert Frost

The CNS is an immune-privileged site. But, the privilege is not absolute and can be disrupted. To immune cells, the CNS endothelium is usually a barrier, but it becomes a beacon when the endothelial cells are inflamed. CNS endothelial cells specifically express the ETX receptor, MAL, required for biologic activity. ETX induces BBB permeability in vivo, causing focal permeability and even frank cerebral edema. Could *C*. *perfringens* ETX affect gut or CNS vascular barriers in MS?

**Figure 1:**
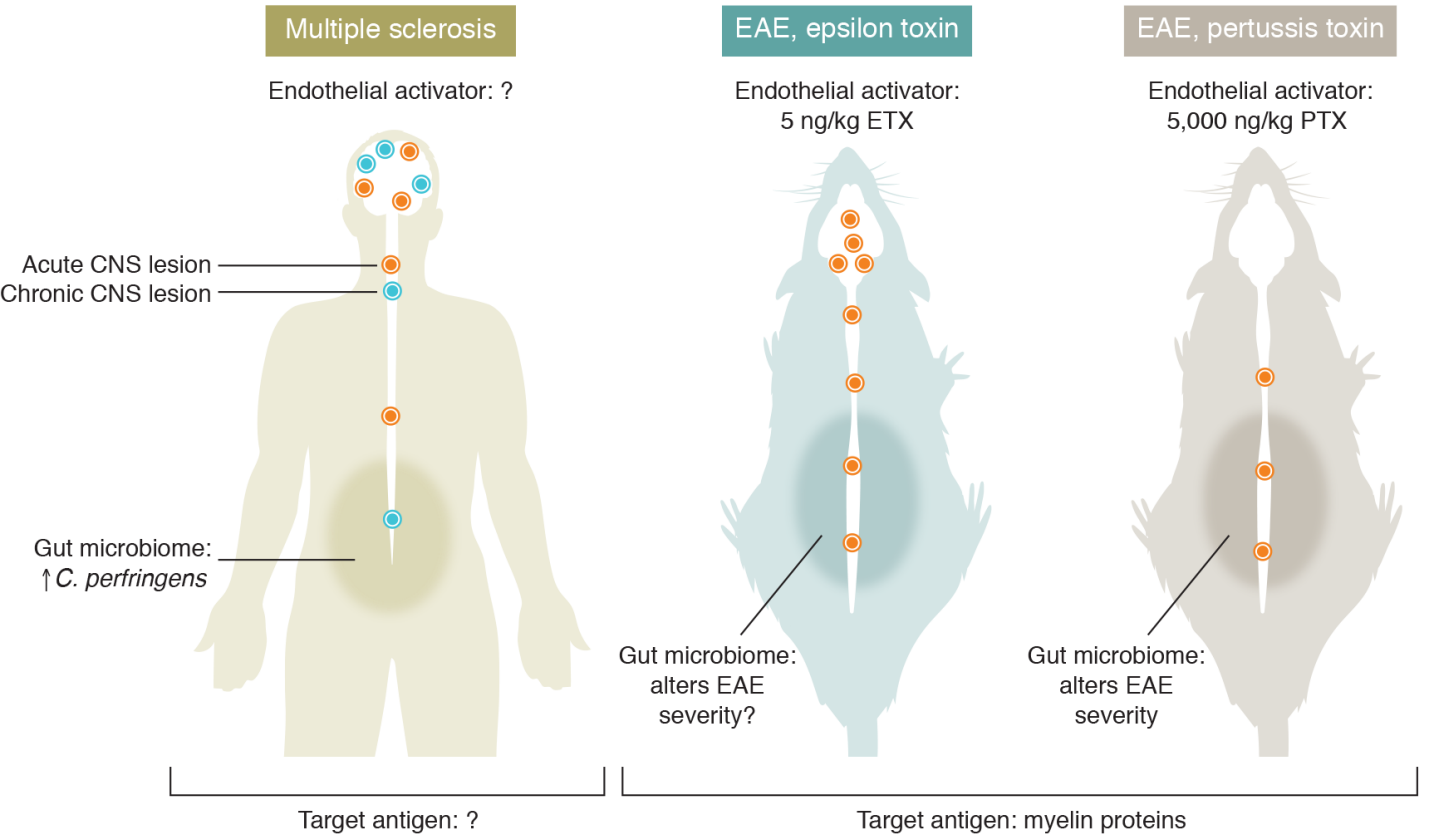
*Clostridium perfringens* epsilon toxin modifies brain lesions in EAE and could affect MS. Patients with MS are more likely to carry two strains of intestinal *Clostridium perfringens*, known to produce ETX. EAE enhanced by ETX shares more similarities to MS pathology than an EAE model that uses the more standard PTX as an endothelial activator. ETX-EAE is characterized by acute lesions in the brain in addition to the spinal cord; whereas PTX-EAE displays acute lesions exclusively in the spinal cord. Fecal transplants from MS patients will worsen EAE, supporting a model whereby pathogenic bacteria from the small intestine promote brain inflammation in MS.

The mechanism by which ETX perturbs CNS inflammation was studied with EAE (9). EAE was induced in female mice by active immunization with the brain antigen, myelin oligodendrocyte glycoprotein peptide (MOG__35-55__), plus oil, *Mycobacterium tuberculosis* adjuvant to enhance antigen presentation, and intraperitoneal *pertussis* toxin (PTX) or ETX to destabilize BBB endothelial cells. PTX actuates ADP-ribosylation of G-proteins in CNS endothelial cells, while ETX and MAL together create pore-forming channels in the endothelial cells. ETX induced disease with a greater semblance to MS than other current EAE models. With PTX, EAE lesions were restricted to the spinal cord, while, with ETX, CD4^^+^^ lymphocytes and CD68^^+^^ microglia appeared in MS-like multifocal lesions of the thalamus, corpus callosum, and cerebellum, causing ataxia in addition to weakness. Clinical EAE appeared with 5,000 ng/kg PTX but with as little as 5 ng/kg ETX (Figure 1). To study direct effects on CNS vasculature, endothelial cells were isolated from spinal cords and brains after two daily injections of PTX or ETX. The two toxins induced largely similar expression of genes that surmount CNS immune privilege, including genes encoding transcription factors, signal transduction factors, inflammatory cytokines, and proteases. ETX uniquely induced *AdamTs8*, *Adamtsl1*, *Pla2g7*, *Mmp2*, and *Lamb1*. These genes encode proteins related to integrin binding, endothelial cell penetration, inflammation, atherosclerosis, COVID-10, BBB degradation, and basement membrane integrity. They could govern focal disruption of the BBB and are potential therapeutic targets in MS.

EAE is a useful tool for studying complex brain-immune interactions and therapies but has pitfalls in extrapolation to MS (4). Only 50% of therapies that are effective in EAE have benefit in MS. Several differences between mice and humans are relevant. In particular, human lymphocytes, but not mouse lymphocytes, express MAL, the ETX receptor. Direct effects of ETX on human immune cells are unknown, and moreover, would be missed in mouse models. MAL gene expression is 3-fold lower in untreated MS than in IFN-β–treated RRMS PBMC (2), possibly due to T cell activation state or loss of immune subsets. There are also differences in the gut function between mouse models and people with MS. Notably, 81% of untreated RRMS, but only 28% of people in the healthy control group, have reduced transport of mannitol across the gut wall, suggesting dysfunctional gut endothelial cells in MS (12). Acute endothelial damage in murine EAE will not reflect the chronic denervation of the gut in MS. Two of three patients with MS showed disintegration of enteric nervous system plexi, compared with luxuriant plexi in 17 people in the healthy control group and one patient with MS (13). Sympathetic input to the GI system and spleen is also disrupted, especially in progressive MS. Sympathetic denervation unleashes a positive feedback loop between inflammation, CNS lesions, and SNS disruption, seen clinically with abnormal sympathetic skin responses, cold purple feet, and constipation (14, 15). Long-term CNS-gut memory is possible. Discrete regions of the insular cortex are topographically paired with inflamed regions of the colon in experimental colitis; weeks later, stimulation of the insula reactivates gut immunity (16). Germ-free and even clean versus dirty environments with broad bacterial colonization will modify the severity of EAE (17). Humans are not germ-free. Unlike healthy antibiotic-free mice, the human microbiome will be altered by antibiotics for treatment of bladder infections, disease-modifying therapies used to treat MS (11, 10), and glucocorticorticoids used to treat MS exacerbations, which could evoke bacterial stress responses and toxin secretion (18).

## Remaining questions and future directions

Why are *C*. *perfringens* strains B and D that produce ETX so common in MS? Is this exuberance linked to changes in other bacterial species or other toxins? Strains B and D are found in ruminants—cows, sheep, goats. Nonetheless, MS is less common in rural areas than in cities, suggesting that additional factors are required to induce MS pathology. Do antibiotics or MS therapies eliminate or affect these strains? Should ETX levels be monitored in clinical trials of MS therapeutics? Does ETX cause MS lesions and fulfill molecular Koch’s postulates of toxin/clinical association, inactivation with loss of ETX, and reinstitution with replacement of the ETX gene (19)? How much dsybiosis is required; how much ETX crosses the gut membrane; and what concentration of ETX in serum and CSF correlates clinically with lesions and EC disruption? PTX and ETX exhibit regional CNS differences in breaching the BBB. ETX seems to target regions with leaky blood vessels (20). Are there regional BBB endothelium hot spots with more expression of MAL and related adhesion molecules? What is the role of MAL in immunity? ETX is produced intermittently, peaking at the end of log-phase bacterial growth, suggesting the importance of bacterial quorum sensing and response to inflammatory stress such as IFN-γ (18). What triggers periodic inflammation or progression in MS and in neuromyelitis optica during chronic colonization with *C*. *perfringens*? Exposure to ETX, at an unknown threshold level, is likely to require additional genetic, dietary, and environmental provocation to cause onset of MS or MS activity. For instance, first Epstein-Barr infection in army recruits is strongly linked to the onset of MS (7). Could ETX be a cofactor or an independent risk factor for MS and other inflammatory and CNS diseases?

## Version 1 05/01/2023

Electronic publication
